# Recent Zoonotic Spillover and Tropism Shift of a Canine Coronavirus Is Associated with Relaxed Selection and Putative Loss of Function in NTD Subdomain of Spike Protein

**DOI:** 10.3390/v14050853

**Published:** 2022-04-21

**Authors:** Jordan D. Zehr, Sergei L. Kosakovsky Pond, Darren P. Martin, Kristina Ceres, Gary R. Whittaker, Jean K. Millet, Laura B. Goodman, Michael J. Stanhope

**Affiliations:** 1 Institute for Genomics and Evolutionary Medicine, Temple University, Philadelphia, PA 19122, USA; jordan.zehr@temple.edu (J.D.Z.); spond@temple.edu (S.L.K.P.); 2 Computational Biology Division, Department of Integrative Biomedical Sciences, Institute of Infectious Diseases and Molecular Medicine, University of Cape Town, Observatory, Cape Town 7549, South Africa; darrenpatrickmartin@gmail.com; 3 Department of Public and Ecosystem Health, Cornell University, Ithaca, NY 14853, USA; kc649@cornell.edu (K.C.); gary.whittaker@cornell.edu (G.R.W.); laura.goodman@cornell.edu (L.B.G.); 4 Department of Microbiology and Immunology, Cornell University, Ithaca, NY 14853, USA; 5 Unité de Virologie et Immunologie Moléculaires, UVSQ, INRAE, Université Paris-Saclay, 78350 Jouy-en-Josas, France; jean.millet@inrae.fr; 6 Baker Institute for Animal Health, Cornell University, Ithaca, NY 14850, USA

**Keywords:** relaxed selection, coronavirus tropism shift, canine coronavirus, feline coronavirus

## Abstract

A canine coronavirus (CCoV) has now been reported from two independent human samples from Malaysia (respiratory, collected in 2017–2018; CCoV-HuPn-2018) and Haiti (urine, collected in 2017); these two viruses were nearly genetically identical. In an effort to identify any novel adaptations associated with this apparent shift in tropism we carried out detailed evolutionary analyses of the spike gene of this virus in the context of related *Alphacoronavirus* 1 species. The spike 0-domain retains homology to CCoV2b (enteric infections) and Transmissible Gastroenteritis Virus (TGEV; enteric and respiratory). This domain is subject to relaxed selection pressure and an increased rate of molecular evolution. It contains unique amino acid substitutions, including within a region important for sialic acid binding and pathogenesis in TGEV. Overall, the spike gene is extensively recombinant, with a feline coronavirus type II strain serving a prominent role in the recombinant history of the virus. Molecular divergence time for a segment of the gene where temporal signal could be determined, was estimated at around 60 years ago. We hypothesize that the virus had an enteric origin, but that it may be losing that particular tropism, possibly because of mutations in the sialic acid binding region of the spike 0-domain.

## 1. Introduction

The ongoing coronavirus (CoV) disease (COVID-19) is the third documented animal to human CoV spillover to have resulted in a major epidemic. Coronaviruses (CoVs) that infect mammals (with the exception of pigs) belong principally to two genetic and serologic groups: the *Alphacoronavirus* (α) and *Βetacoronavirus* (β) genera. *Alphacoronavirus* 1, a species within the *Alphacoronavirus* genus that infects dogs, cats and pigs, is further subdivided into type I and II, and is typically associated with gastroenteritis. Vlasova et al. [1] recently reported on an *Alphacoronavirus* 1 CoV resembling Canine CoV (CCoV; named CCoV-HuPn-2018), isolated from nasopharyngeal swabs of a small number of pediatric patients (8 of 301) in Sarawak, Malaysia, hospitalized with pneumonia between 2017 and 2018. CCoV-HuPn-2018 resembles a CCoV type II, but also shares high nucleotide sequence similarity with other type II *Alphacoronavirus* 1 CoVs: feline CoV (FCoV2) and porcine Transmissible Gastroenteritis Virus (TGEV). Subsequently to the original Vlasova et al. report, there was identification of a genetically similar virus (99.4% identical across the genome, compared to CCoV-HuPn-2018) from the urine samples of a medical worker returning from Haiti who was experiencing mild fever and malaise [2], named HuCCoV_Z19Haiti, suggesting that human infection with this CCoV virus may have occurred in multiple locations. Both studies report on genome-wide recombination history of the virus, implicating a FCoV type ll virus as a significant contributor. Very recently, a cryoEM structure determination of the spike protein of CCoV-HuPn-2018 was released as a preprint [3].

A key determinant of CoV tissue tropism and host range is the ability of the spike protein to bind with host cellular receptors. The spike protein is responsible for host receptor binding and fusion of the virus with host cell membranes [4]. It is comprised of the N-terminal S1 region, containing the receptor binding domain (RBD), and the C-terminal S2 region, responsible for membrane fusion and cellular entry. The CCoV receptor in dogs, and for the rest of the type II members of the *Alphacoronavirus* 1 species, is amino peptidase N (APN) (reviewed in Millet et al. [5]). The human common-cold coronavirus, HCoV-229E, another *Alphacoronavirus* (not *Alphacoronavirus* 1), also uses APN. Structural studies involving porcine respiratory coronavirus (PRCV; a variant of TGEV) indicate that it binds to a site on the porcine APN that differs from the site at which HCoV-229E binds to hAPN [6,7], implying that there are multiple ways for this interaction to take place. Importantly, feline APN can serve as a functional receptor of type II CCoV, TGEV and human coronavirus HCoV-229E [8]. While definitive experimental data are still lacking, the possibility of co-infections in cats implies that individual cells can become infected with these different CoVs, which could, in turn, generate novel recombinant strains. Spike gene recombination has played an important role in the evolution of the *Alphacoronavirus* 1 type II CoVs, involving recombination between dog, cat and pig viruses, including the complete replacement of the most N-terminus subdomain of CCoV2 with that of TGEV—an important event in the formation of CCoV2b [9].

In addition to APN, *Alphacoronavirus* 1 CoVs have been reported to use other co-receptors, including C-type lectins, dendritic cell-specific intercellular adhesion molecule-3-grabbing non-integrin (DC-SIGN), heparan sulfate (HS) and sialic acid (reviewed in Millet et al. [5]). Thus, there are numerous possible avenues for developing new receptor interactions, an important step in cross-species transmission. Sialic acid binding activity has been confirmed for TGEV [10] and point mutations or a short deletion near the N-terminus of the spike protein have been shown to abrogate binding to this co-receptor, as well as result in lower viral pathogenicity [11]. PRCV, a naturally occurring variant of TGEV, typifies how a modification in the N-terminal region of S1 can have a profound impact on tissue tropism. The S1 of PRCV has lost its N-terminal region through a deletion which resulted in the virus losing its sialic acid binding ability (reviewed by Hulswit et al. [12]) and this loss of function modified the tissue tropism of PRCV to predominately respiratory.

Here, we provide a comprehensive analysis of the selective pressures across the spike gene, focusing on protein functional domains and possible implications with regard to this apparent viral tropism shift. We also supplement the existing information on the recombination history of the virus by analyzing sequence specific contributors and putative donor-recipient relationships within the spike gene.

## 2. Materials and Methods

### 2.1. Sequences and Alignments

We collected all available complete spike gene sequences from *Alphacoronavirus* 1 type II CoVs available in GenBank (accession numbers appear in Supplementary Table S1). Spike sequences from experimental inoculations, along with partial sequences, were excluded from the analysis, since experimental strains do not represent naturally replicating viruses, and partial sequences are not informative for recombination analyses—a precursor to selection inference. CCoV type II viruses are currently split into two groups: CCoV2a and CCoV2b. Our choice of CCoV2b as our representative CCoV from the dog host is informed by the fact that the most N-terminal subdomain (0-domain) of the spike protein of CCoV2b and TGEV are homologous to CCoV-HuPn-2018, whereas this is not the case for either CCoV2a or FCoV2. We were not able to include more members of the *Alphacoronavirus* genus in our analyses because the divergence of other species compared to *Alphacoronavirus* 1 precludes reliable codon-aware sequence alignments. When unreliable alignments are analyzed for evidence of natural selection, high rates of false positives are common [13]. CCoV-HuPn-2018 and HuCCoV_Z19Haiti have an almost identical spike gene sequence, with one synonymous and two nonsynonymous changes, and no insertion or deletions. Our analyses were completed on CCoV-HuPn-2018 prior to the release of the HuCCoV_Z19Haiti sequence, but to verify the selection pressure signal involving these two, we repeated analyses with HuCCoV_Z19Haiti; no differences were noted. All analyses herein reported involve the CCoV-HuPn-2018 sequence. Spike domain site positional mapping is based on the spike gene map from Tortorici et al. [3].

We prepared two sets of alignments for comparative sequence analyses. Alignment set I (the 5′ end of the spike gene up to and including codon 266 of CCoV-HuPn-2018) was assembled to analyze the 0-domain [3] and includes TGEV, CCoV-HuPn-2018, and CCoV2b strains. The second set (alignment set II) included all of the above strains, as well as FCoV2, but now involved positions downstream of codon CCoV-HuPn-2018 266, which is the onset of the region where FCoV2 and the other sequences are homologous. Type I and type II *Alphacoronavirus* 1 viruses were not included together in sequence alignments used for selection analyses, because the divergence between these two types does not yield a reliable nucleotide alignment. In-frame nucleotide sequences were translated to amino-acids, aligned with MAFFT [14], and then mapped back to the nucleotide sequences to produce a codon-aware alignment.

### 2.2. Recombination, Positive Selection, Temporal Dating

Each of alignment sets I and II were screened for genetic recombination using two different approaches. Firstly, we used GARD [15] to search for the optimal number and location of recombination breakpoints in an alignment, based on the AIC-c information theoretic criterion. GARD is powered by major alterations to the tree topologies, i.e., alterations which are large enough to statistically justify the inclusion of multiple trees over the gene alignment (2N-3 parameters per tree, where N is the number of sequences). Molecular selection analyses can be confounded if a single tree is used to analyze alignments with a strong recombination signal, i.e., where different trees are supported by different parts of the gene alignment, typically resulting in higher rates of false positives [15]. Pre-screening alignments with GARD allows for accurate selection inference in the presence of recombination, for each of the partitions defined by the GARD breakpoints. A maximum likelihood phylogeny was inferred for each partition using RAxML [16] under the GTR + Γ nucleotide substitution model. Each codon-aware alignment partition concomitant with their respective inferred phylogeny served as an input to the selection analyses. Secondly, we used RDP5 [17] to identify sequence-specific contributors to recombination events, and to identify putative donor/recipient pairs for each event. More specifically, we identified recombination events that explicitly implicate CCoV-HuPn-2018, and considered the event well-supported if 4 of the 7 methods implemented in RDP5 (RDP, GENECONV, Bootscan, Maxchi, Chimaera, SiScan, and 3seq) were significant for detecting the event (*p* < 0.05).

We performed site- and branch-level selection tests based on the dN/dS (nonsynonymous/synonymous) rate ratio estimation as implemented in the HyPhy software package v.2.5.32 [18] to test for evidence of positive selection acting on sites within the CCoV-HuPn-2018 sequence, as well as evidence of positive selection on the CCoV-HuPn-2018 terminal branch (a subset of sites along this branch) in each phylogeny. We used MEME [19] to detect episodic diversifying selection pressure and FEL [20] to detect purifying and positive pervasive diversifying selection at individual sites in CCoV-HuPn-2018 for each GARD partition, with its respective ML phylogeny. For this study, we modified the MEME and FEL tests to use a parametric bootstrap for estimating the null distribution of the likelihood ratio test instead of the asymptotic distribution used in the published tests. This was done to improve the statistical performance of the tests when the test set comprises a single branch, with the tradeoff of a significant increase in computational cost. We used 100 parametric bootstrap replicates to generate the distribution of the likelihood ratio (LR) test statistic under the null hypothesis (neutral evolution or negative selection). Positively selected sites were mapped to 3D protein structures determined using AlphaFold2 [21], implemented in ColabFold [22], for the 0, A and B domains [3] of the CCoV-HuPn-2018 spike protein. We also tested the CCoV-HuPn-2018 terminal branch for evidence of episodic positive diversifying selection (some subset of sites along this branch), using the aBSREL [23] and BUSTED [24] methods. Lastly, we compared the strength of selection acting on the CCoV-HuPn-2018 sequence relative to TGEV, CCoV2b and FCoV2 sequences with the RELAX method [25].

To date the possible origin of CCoV-HuPn-2018, we employed state of the art procedures in molecular clock dating analysis. The temporal signal in each GARD partition was assessed using root-to-tip regression in TempEst v1.5.3 [26] and tip-dating-randomization tests (TDR) [27]. Each phylogenetic tree (IQTREE-2 [28]) with the best-fitting substitution model (ModelFinder [29]) was used as input for root-to-tip regression analysis. For GARD partitions with a correlation coefficient greater than 0.1, temporal signal was confirmed using TDR. The R package TipDatingBeast [30] was used to generate ten random permutations of sample dates for each GARD alignment. BEAST2 [31] was then used to estimate the evolutionary rate for both alignments with the true sample dates and alignments for each randomized replicate. If the mean clock rate estimate of the alignment with real sample dates fell outside the 95% highest posterior density (HPD) for the randomized date set, temporal signal was deemed sufficient for subsequent analyses.

For each alignment that had sufficient evidence of a temporal signal, the fit of combinations of two molecular clock models (strict and uncorrelated relaxed exponential [32]) and two demographic models (constant coalescent and Bayesian skyline plot [33]) were assessed using marginal likelihood estimation (PathSampling [34]). The average marginal likelihood estimates from two path sampling runs were compared to other model combinations using Bayes Factors [35]. The ancestral state of the host species (cat, dog, pig, human) was inferred using discrete ancestral trait mapping in BEAST2 [31] and Tracer v1.7.1 [36]. More detailed method descriptions of protein structure mapping and temporal dating appear in Supplementary Material.

## 3. Results

### 3.1. Recombination and Temporal Dating

**Figure 1:**
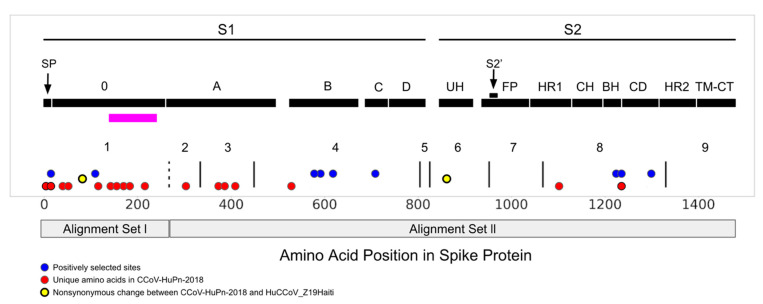
Positive selection, unique amino acid changes and GARD partitions mapped to a CCoV-HuPn-2018 spike domain map [3]. S1 and S2 of the protein are highlighted and further subdivided into functional subunits and subdomains. Blue dots represent sites under positive selection in CCoV-HuPn-2018 as identified by MEME and/or FEL; red dots represent sites that are unique in CCoV-HuPn-2018 but are not under positive selection; yellow dots are nonsynonymous changes between CCoV-HuPn-2018 and HuCCoV_Z19Haiti. Text labels accompany each subdomain/functional unit: SP, signal peptide; 0 domain; A domain; B, includes RBD-Receptor-Binding Domain; C; D; UH, upstream helix; FP, fusion peptide; HR1, heptad-repeat 1; CH, central helix; BH, β-hairpin; CD: connector domain; HR2, heptad-repeat 2; TM, transmembrane domain; CT, cytoplasmic tail. The horizontal magenta bar represents the experimentally evaluated region for sialic acid binding in TGEV [11,37]. The solid vertical black lines represent the breakpoints of the GARD identified non-recombinant fragments and are labeled numerically. The vertical dashed line represents the 3′ end of alignment set I and the onset of FCoV2 sequence homology (alignment set II).

The GARD [15] method did not detect any recombination in the 0-domain (alignment set I); we consider it as GARD partition 1. This non-recombinant partition does not include FCoV2 because it is located upstream of the beginning of the region of FCoV2 sequence homology with the rest of the sequences in our analysis (Figure 1; see Methods for a description of rationale and composition of these two alignment sets). GARD inferred eight putatively non-recombinant partitions for alignment set II, and their corresponding ML phylogenies can be found in Supplementary Figure S1.

**Figure 2:**
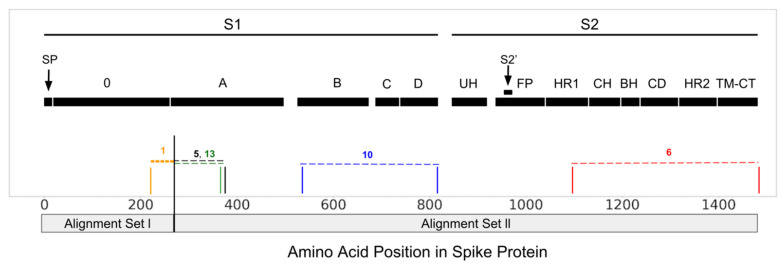
RDP5 [17] results with supported recombination events (event boundaries outlined) that implicate CCoV-HuPn-2018, positioned along the same spike domain map as Figure 1 [3].

We also ran RDP5 [17] on both alignment sets to determine which sequences were recombinant and to identify likely parental sequences; the complete set of RDP5 results appears in Supplementary Table S2. In alignment set I, RDP5 identified one event (denoted event 1) that was well-supported (see methods section for more details), with the breakpoint at amino acid position 223 continuing downstream to the end of the alignment, but without a clearly identifiable donor/recipient relationship. Alignment set ll contained four well-supported events that directly implicate the CCoV-HuPn-2018 sequence (Figure 2). Two of the four well supported events identify CCoV-HuPn-2018 as the major donor, with an FCoV2 minor donor and FCoV2 recombinant, and a CCoV2b minor donor and CCoV2b recombinant, for events 5 and 6, respectively. The other two well-supported events identify CCoV-HuPn-2018 as the proposed recombinant, with an FCoV2 major donor and unknown minor donor, and an unknown major donor and a TGEV minor donor for events 10 and 13, respectively. Recombination events 5 and 13 occur at the 5′ end of the A-domain and had predicted breakpoint locations that overlapped almost exactly with those of GARD partition 2. Event 10 falls in the middle of alignment set ll, encapsulating domains B through D and roughly matching up with GARD partition 4. Event 6 falls at the 3′ end of alignment set ll and overlaps with GARD partitions 8 and 9. Events 5 and 10 both implicate CCoV-HuPn-2018 and a FCoV2 strain WSU 79-1683 (accession number JN634064), isolated at Washington State University in 1979 [38].

GARD partition 7 was the only partition with substantive temporal signal in the root-tip-regression and TDR (Tip-Dating-Randomization) analyses (Supplementary Table S3, and Supplementary Figure S2). BEAST2 [31] analysis of GARD partition 7 suggested CCoV-HuPn-2018 may have diverged from a lineage most recently circulating in cats between 1846 and 1976 (95% HPD—Highest Posterior Density Interval), with a median estimate of 1957 (Supplementary Figure S3).

### 3.2. Selection Pressure

In analyses involving MEME [19] and FEL [20], we found moderate statistical evidence of positive selection acting upon individual sites in various regions of the CCoV-HuPn-2018 spike gene (Figure 1; statistics summarized in Supplementary Table S4; positively selected sites mapped to 3D protein structures for the 0, A and B domains appears in Supplementary Figure S4). RBD (Receptor Binding Domain; B-domain) extended loops form the interaction points with the APN receptor in several Alphacoronaviruses [7,39,40]. Tortorici et al. [3] report that the B-domain structure of CCoV-HuPn-2018 is very similar to PRCV, including key APN interacting residues (Y543/W586). One of four B-domain positively selected sites (Figure 1) was in this same interacting loop at position 575 (Supplementary Figure S4).

There were no statistically significant results for aBSREL [23] and BUSTED [24]. The 0-domain of CCoV-HuPn-2018 (GARD partition 1; Figure 1) had eight unique amino acid residues (not inferred to be under positive selection) relative to the CCoV2b and TGEV sequences, as well as one site inferred to be evolving under positive selection. The signal peptide had one site under positive selection and two additional unique amino acid changes. The 0-domain of the CCoV-HuPn-2018 NTD comprises 243 residues with homology only to available TGEV and CCoV2b isolates. Earlier studies have reported on the similarity involving this latter pair, implicating recombination between CCoV2 and TGEV [9,41] in the origins of CCoV2b [41]. The 0-domain of CCoV-HuPn-2018 was subject to relaxed selection compared to other CCoV and TGEV sequences (GARD partition 1; RELAX [23] results: K = 0.07; *p* = 0.007 for CCoV2b vs. CCoV-HuPn-2018 and K = 0.081; *p* = 0.04 for TGEV vs. CCoV-HuPn-2018; for an additional level of detail regarding the contributions of these two viruses to this relaxed signal, see Supplementary Figure S5). CCoV-HuPn-2018 also had an increased rate of molecular evolution relative to the other CCoV2b and TGEV sequences (significant Tajima’s relative rate test, implemented in MEGA X [42]; all three codon positions, as well as synonymous alone, and for most nonsynonymous pairwise comparisons).

### 3.3. Sialic Acid Binding Region

**Figure 3:**
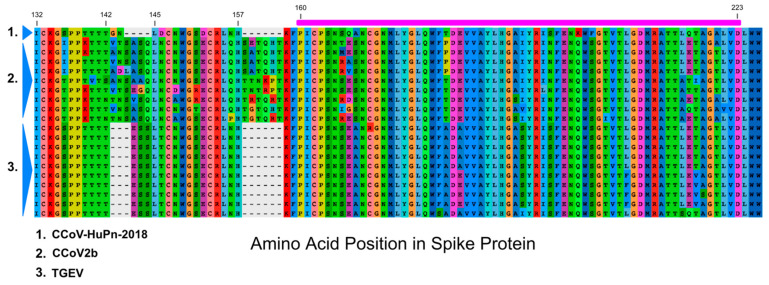
Amino acid sequence alignment of the downstream region of 0-domain with magenta shaded bar above the alignment highlighting the region experimentally evaluated as relevant to sialic acid binding in TGEV [11,37]; numbers correspond to unaligned positions in the CCoV-HuPn-2018 spike protein.

Krempl et al. [11,37] identified a 3′ portion of the 0-domain sequence in which either point mutations or a short deletion eliminates sialic acid binding in TGEV and are associated with lower viral pathogenicity. This sialic acid binding region overlaps with a region in alignment set I that has several unique amino acid changes in CCoV-HuPn-2018 (Figure 1 and Figure 3), as well as an RDP5 identified recombinant region (Figure 2). Immediately upstream of the region in the Krempl et al. experiments, in our alignment set I, was an orthologous deletion between CCoV-HuPn-2018 and TGEV and a unique indel involving all three virus types (Figure 3).

## 4. Discussion

Spike NTD domains in CoV are being increasingly recognized for their role in infection. The NTD may act as a co-receptor binding domain for SARS-CoV-2, interacting with the tyrosine-protein kinase receptor UFO (AXL [43]) and possibly sialic acids (reviewed in Sun et al. [44]). Sialic acid binding in the NTD has been confirmed for TGEV and porcine epidemic diarrhea virus (PEDV) [10,45]. Similar experimental data are not available for CCoV2bs; however, both pigs and dogs are known to have the same sialic acid receptors present on cells from similar anatomical regions [46]. The precise role of sialic acid binding in TGEV remains uncertain, with the possibility that it functions either as a co-receptor or protects the virus in the hostile conditions of the digestive tract [12]. Krempl et al. [37] have suggested that their experimentally mutated sites may play a structural role in sialic acid binding, rather than directly interacting with the sialic acid, suggesting that not only the specific sites, but adjacent sequence regions, such as the gapped sequence illustrated in alignment Figure 3, could be at play in this overall interaction. The complete absence of the 0-domain in PRCV eliminated sialic acid binding [47], altered the pathogenicity of PRCV, and switched its predominant tropism to the respiratory tract [11]. TGEV, on the other hand, can infect both the respiratory and enteric tracts [48]. Although it is unclear from [2] whether HuCCoV_Z19Haiti was a respiratory infection, its isolation from urine samples does not preclude this, since other respiratory CoVs, such as SARS-CoV-2, can be detected in urine [49]. A human respiratory *Alphacoronavirus*—HCoV-229E—does not contain the 0-domain and may have originated from bat coronaviruses with intestinal tropism that do possess this sub-domain [50].

Another region of note with regard to unique changes and positive selection was the CCoV-HuPn-2018 signal peptide (Figure 1). Many viruses make use of the host cell process of N-linked glycosylation to modify surface proteins, including the spike of CoVs [39], and this can impact antigenicity and host cell invasion. Recent work on HIV found that the signal peptide can influence the glycan profile and antigenicity of the HIV surface protein gp120 [51], prompting these authors to suggest that despite the fact the signal peptide is not part of the mature protein, it is likely to be subject to immune pressure. Positive selection within signal peptides has been reported for other viruses, such as cytomegalovirus, where the selected variants affect the timing of signal peptide removal and viral glycoprotein intracellular trafficking [52]. We propose that positive selection in the signal peptide of CCoV-HuPn-2018 could reflect an adaptive role in this new host.

The above results and prior studies lead us to the hypothesis that CCoV-HuPn-2018 had an enteric origin (based on its similarity to dog, cat and pig *Alphacoronavirus* 1), but has lost that particular tropism, possibly due in part to mutations and/or recombination in the sialic acid binding region of the 0-domain, resulting in the loosening of functional constraints, ultimately reflected in the relaxed selection pressure identified in our analyses. Alternatively, the virus may have infected a different host, such as a human, where the 0-domain was no longer fulfilling the same role, and/or simply could not interact with the carbohydrates of its new host, again resulting in relaxed selection pressure. Analogous to other *Alphacoronavirus* 1 lineages that have lost the 0-domain and developed an alternative tissue tropism (e.g., PRCV), we may be witnessing this process in transition for CCoV-HuPn-2018. Based on the single portion of the spike gene that exhibited temporal signal, the timing of the origins of CCoV-HuPn-2018 to approximately 1957, and the co-occurrence of the same (or very similar) virus in Haiti that harbors additional recombinant portions in the 3′ end of the genome [2], suggest that this virus may have been circulating undetected in humans, dogs, cats or intermediate unidentified hosts for decades.

## Data Availability

All data used in this study are publicly available on NCBI (https://www.ncbi.nlm.nih.gov/, accessed on 29 November 2021). A list of the accession numbers used is found in Supplementary Table S1.

## Supplementary Materials

The following supporting information can be downloaded at: https://www.mdpi.com/article/10.3390/v14050853/s1, Figure S1: GARD partition phylogenies; Table S1: Accession numbers of complete, naturally occurring infection Spike sequences used in this analysis; Figure S2: TDR clock rate estimates; Table S2: RDP5 results for alignment sets 1 and 2; Figure S3: Ancestral host reconstruction and divergence time estimates; Table S3: Root-tip-regression results for each GARD partition; Figure S4: Positively selected and unique sites mapped to 0, A, and B domains using AlphaFold2 3D structural predictions; Table S4: Sites under positive selection in CCoV-HuPN-2018 as identified by MEME and FEL; Figure S5: Relaxed selection results for 0-domain.
