# Supplementary material for: Recent Zoonotic Spillover and Tropism Shift of a Canine Coronavirus Is Associated with Relaxed Selection and Putative Loss of Function in NTD Subdomain of Spike Protein
Source: Viruses. 2022 Apr 21;14(5):853. doi: 10.3390/v14050853 (PMC9145938; doi:10.3390/v14050853)
Supplement: Supplementary file 1 [file viruses-14-00853-s001.zip › VIRUSES-Apr20-Suppl-Methods.pdf]

SUPPLEMENTARY METHODS

Alphafold2 and Mapping/ Visualization

Domains 0, A, and B, as determined by the domain map provided by Tortorici et al [1], were used as input for 3D structural prediction. Structural prediction was performed with AlphaFold2 [2], through a publicly available version of the software implemented with ColabFold [3]. Each prediction was performed with default parameters. For each predicted structure, two metrics were output: Predicted Local Distance Difference Test (pLDDT) and Predicted Aligned Error (PAE).

Residues within each structurally predicted PDB were annotated according to selection results from MEME [4] and FEL [5], if the site was unique compared to TGEV and CCoV2b, and if the site was a non-synonymous change between CCoV-HuPn-2018 and HuCCoV_Z19Haiti, blue, red, and yellow, respectively (Supplementary Figure S4). Observable Notebook (https://observablehq.com) was used to visualize the annotated PDB structures, and can be found here: https://observablehq.com/@jzehr.

Estimation of divergence times

Temporal signal

The temporal signal in each GARD partition was assessed using root-to-tip regression in TempEst v1.5.3 [6] and tip-dating-randomization tests (TDR) [7]. First, ModelFinder [8] was used in IQTREE-2 [9] to identify the best fitting substitution model for each alignment using Bayesian Information Criterion (BIC). Each tree with the best-fitting substitution model was then used as input for root-to-tip regression analysis, where correlation coefficients were calculated using the heuristic residual mean squared function. If a strong temporal signal exists (a linear relationship between genetic distance and sampling time), the correlation coefficient will be positive. For GARD partitions with correlation coefficient greater than 0.1, temporal signal was confirmed using TDR. The R package TipDatingBeast [10] was used to generate ten random

permutations of sample dates for each GARD [11] alignment. BEAST2 [12] was then used to estimate the evolutionary rate for both alignments with the true sample dates and alignments for each randomized replicate. If the mean clock rate estimate of the alignment with real sample dates fell outside the 95% highest posterior density (HPD) for the randomized date set, temporal signal was deemed sufficient for subsequent analyses.

Model selection

For each alignment that had sufficient evidence of a temporal signal, the fit of combinations of two molecular clock models (strict and uncorrelated relaxed exponential [13]) and two demographic models (constant coalescent and Bayesian skyline plot [14]) were assessed using marginal likelihood estimation. For each model tested, marginal likelihood was calculated using PathSampling [15] within the Model-Selection package in BEAST 2 with 12 steps, 1,000,000 MCMC steps with 25% burn-in, and an alpha of 0.3. The average marginal likelihood estimates from two path sampling runs were compared to other model combinations using Bayes Factors [16].

Discrete trait analysis

The ancestral state of host species (cat, dog, pig, human) was inferred using discrete ancestral trait mapping in BEAST2 [12] for each GARD [11] alignment. Bayesian phylogenies were created using 100 million MCMC steps in BEAST2, sampling every 10,000 steps. Trees were summarized using the BEAST2 package TreeAnnotator v2.6.0, discarding 20% of trees as burn-in. Convergence of the MCMC chains was assessed using Tracer v1.7.1 [17] and the effective sample size for each estimated parameter was confirmed to be greater than 200. Phylogenetic trees were annotated using FigTree v1.4.4 (available from http://tree.bio.ed.ac.uk/software/figtree/).

References

1. Tortorici, MA.; Walls, AC.; Joshi, A.; Park, Y-J.; Eguia, RT.; Stevens-Ayers, T.; Boeckh, MJ, Telenti, A.; Lanzavecchia, A.; Corti, D.; Bloom, JD.; Veesler, D. Tortorici Structure, receptor recognition and antigenicity of the human coronavirus CCoV-HuPn-2018 spike glycoprotein. *biorxiv.org/content/10.1101/2021.10.25.465646v1* **2021**; DOI:10.1101/2021.10.25.465646.

2. Jumper, J.; Evans, R.; Pritzel, A.; Green, T.; Figurnov, M.; Ronneberger, O.; Hassabis, D. Highly accurate protein structure prediction with AlphaFold. *Nature* **2021**, *596*, 583-589; DOI:10.1038/s41586-021-03819-2.

3. Mirdita, M.; Schütze, K.; Moriwaki, Y.; Heo, L.; Ovchinnikov, S.; Steinegger, M. ColabFold-Making protein folding accessible to all. biorxiv.org/content/10.1101/2021.08.15.456425v2, **2021**.

4. Murrell, B.; Wertheim, JO.; Moola, S.; Weighill, T.; Scheffler, K.; Kosakovsky, Pond SL. Detecting individual sites subject to episodic diversifying selection. *PLoS Genet.* **2012**, *8*, e1002764; DOI:10.1371/journal.pgen.1002764.

5. Kosakovsky, Pond SL.; Frost, SD. Not so different after all: a comparison of methods for detecting amino acid sites under selection. *Mol Biol Evol.* **2005**, *22*, 1208-1222; DOI:10.1093/molbev/msi105.

6. Rambaut, A.; Lam, TT.; Max, Carvalho L.; Pybus, OG. Exploring the temporal structure of heterochronous sequences using TempEst (formerly Path-O-Gen). *Virus Evol.* **2016**, *2*, vew007; DOI:10.1093/ve/vew007.

7. Duchêne, S.; Duchêne, D.; Holmes, EC.; Ho, SY. The performance of the Date-Randomization Test in phylogenetic analyses of time-structured virus data. *Mol Biol Evol.* **2015** *32*, 1895-1906; DOI:10.1093/molbev/msv056.

8. Kalyaanamoorthy, S.; Minh, BQ.; Wong, TKF.; von Haeseler, A.; Jermiin, LS. ModelFinder: fast model selection for accurate phylogenetic estimates. *Nat Methods*. **2017**, *14*, 587-589; DOI:10.1038/NMETH.4285.

9. Minh, BQ.; Schmidt, HA.; Chernomor, O.; Schrempf, D.; Woodhams, MD.; von Haeseler, A.; Lanfear, Robert R. IQ-TREE 2: New models and efficient methods for phylogenetic inference in the genomic era. *Mol Biol Evol*. **2020**, 37, 1530-1534; DOI:10.1093/molbev/msaa015.

10. Rieux, A.; Khatchikian, CE. tipdatingbeast: an r package to assist the implementation of phylogenetic tip-dating tests using beast. *Mol Ecol Resour.* **2017**, *17*, 608-613; DOI:10.1111/1755-0998.12603.

11. Kosakovsky, Pond SL.; Posada, D.; Gravenor, MB.; Woelk, CH.; Frost, SD. GARD: a genetic algorithm for recombination detection. *Bioinformatics* **2006**, 22, 3096-3098; DOI:10.1093/bioinformatics/btl474.

12. Bouckaert, R.; Heled, J.; Kühnert, D.; Vaughan, T.; Chieh-his, Wu C-H.; Xie, D.; Suchard, MA.; Rambaut, A.; Drummond, AJ. BEAST 2: a software platform for Bayesian evolutionary analysis. *PLoS Comput. Biol.*, **2014**, *10,* e1003537; DOI:10.1371/journal.pcbi.1003537.

13. Drummond, AJ.; Ho, SY.; Phillips, MJ.; Rambaut, A. Relaxed phylogenetics and dating with confidence. *PLoS Biol*. **2006**, *4*, e88; DOI:10.1371/journal.pbio.0040088.

14. Drummond, AJ.; Rambaut, A.; Shapiro, B.; Pybus, OG. Bayesian coalescent inference of past population dynamics from molecular sequences. *Mol Biol Evol*. **2005**, *22,* 1185-1192; DOI:10.1093/molbev/msi103.

15. Lartillot, N.; Philippe, H. Computing Bayes factors using thermodynamic integration. *Syst Biol*. **2006**, 55, 195-207; DOI:10.1080/10635150500433722.

16. Kass, RE.; Raftery, AE. Bayes Factors. *J Am Stat Assoc.* **1995**, *90*, 773-795.

17. Rambaut, A.; Drummond, AJ.; Xie, D.; Baele, G.; Suchard, MA. Posterior summarization in Bayesian phylogenetics using Tracer 1.7. *Syst Biol*. **2018**, 67, 901-904; DOI:10.1093/sysbio/syy032.
