# Supplementary material for: Recent Zoonotic Spillover and Tropism Shift of a Canine Coronavirus Is Associated with Relaxed Selection and Putative Loss of Function in NTD Subdomain of Spike Protein
Source: Viruses. 2022 Apr 21;14(5):853. doi: 10.3390/v14050853 (PMC9145938; doi:10.3390/v14050853)
Supplement: Supplementary file 1 [file viruses-14-00853-s001.zip › VIRUSES-Apr20-Supplimental_figs_tableS3.pdf]

Figure S1. GARD partition phylogenies

1.

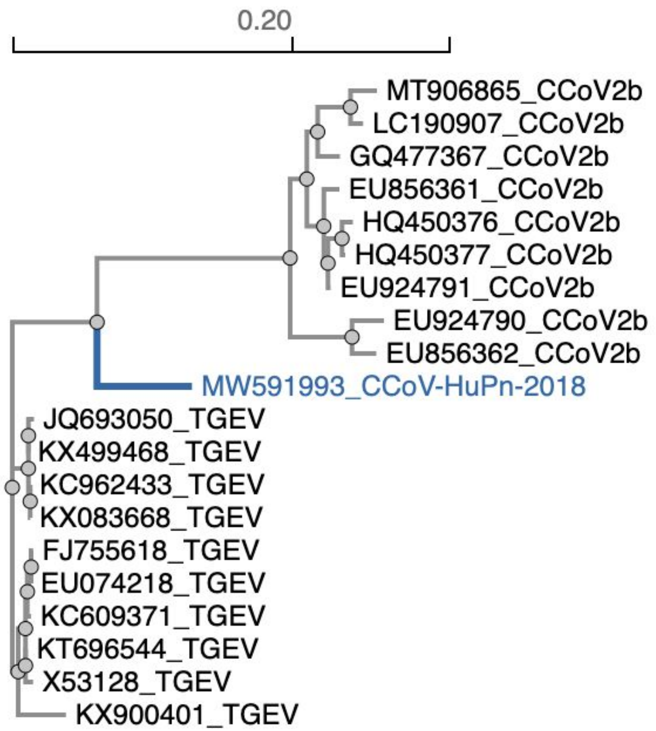

2.

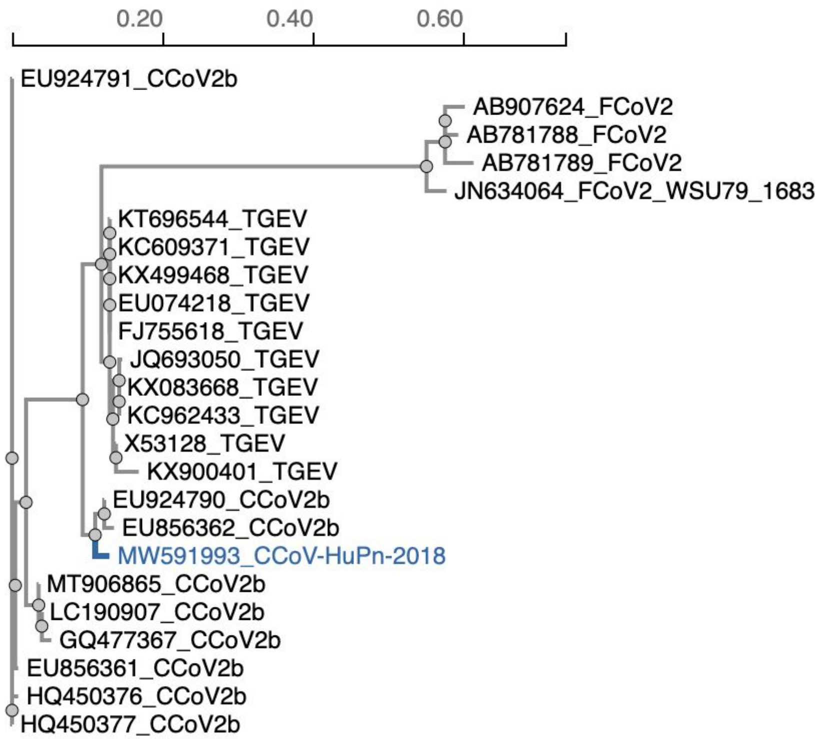

3.

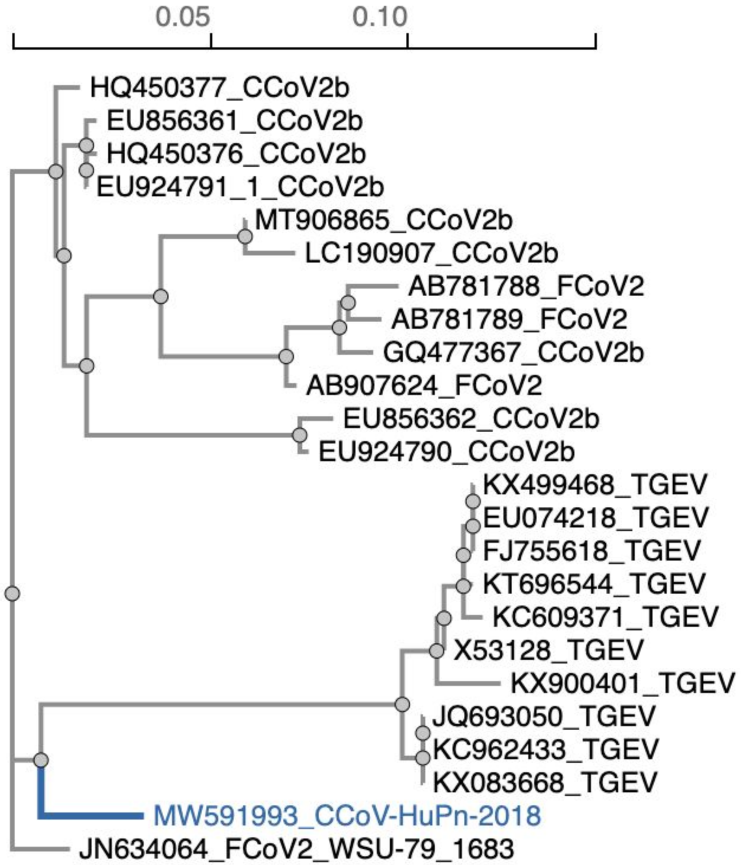
0.05
0.10
HQ450377_CCoV2b
EU856361_CCoV2b
HQ450376_CCoV2b
EU924791_1_CCoV2b
MT906865_CCoV2b
LC190907_CCoV2b
AB781788_FCoV2
AB781789_FCoV2
GQ477367_CCoV2b
AB907624_FCoV2
EU856362_CCoV2b
EU924790_CCoV2b
KX499468_TGEV
EU074218_TGEV
FJ755618_TGEV
KT696544_TGEV
KC609371_TGEV
X53128_TGEV
KX900401_TGEV
JQ693050_TGEV
KC962433_TGEV
KX083668_TGEV
MW591993_CCoV-HuPn-2018
JN634064_FCoV2_WSU-79_1683

4.

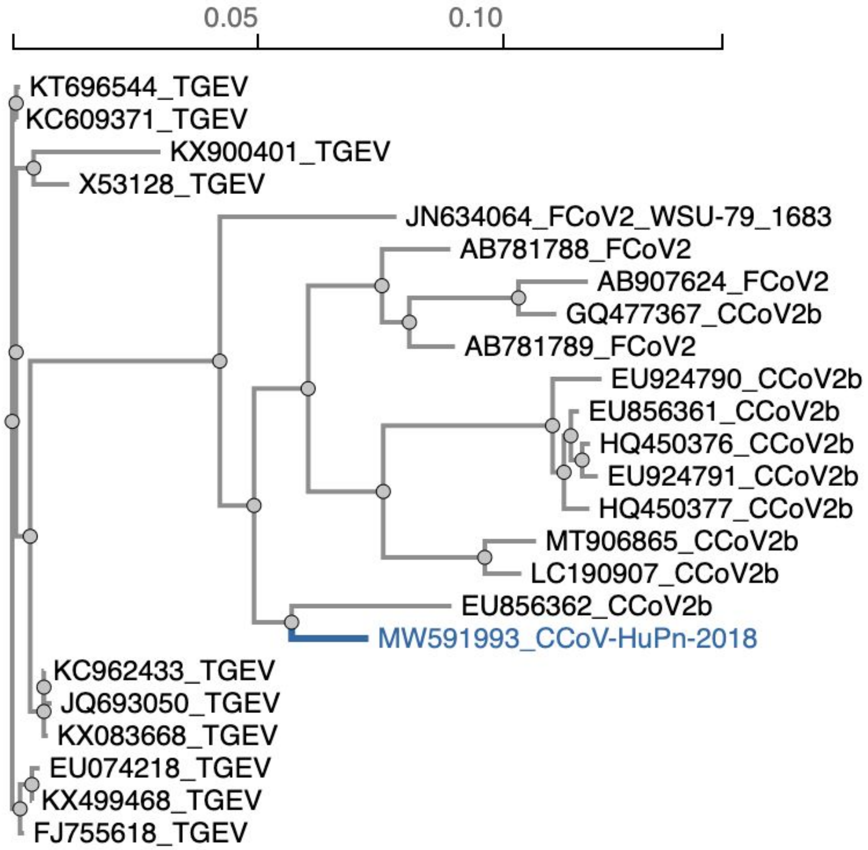
0.05
0.10
KT696544_TGEV
KC609371_TGEV
KX900401_TGEV
X53128_TGEV
JN634064_FCoV2_WSU-79_1683
AB781788_FCoV2
AB907624_FCoV2
GQ477367_CCoV2b
AB781789_FCoV2
EU924790_CCoV2b
EU856361_CCoV2b
HQ450376_CCoV2b
EU924791_CCoV2b
HQ450377_CCoV2b
MT906865_CCoV2b
LC190907_CCoV2b
EU856362_CCoV2b
MW591993_CCoV-HuPn-2018
KC962433_TGEV
JQ693050_TGEV
KX083668_TGEV
EU074218_TGEV
KX499468_TGEV
FJ755618_TGEV

5.

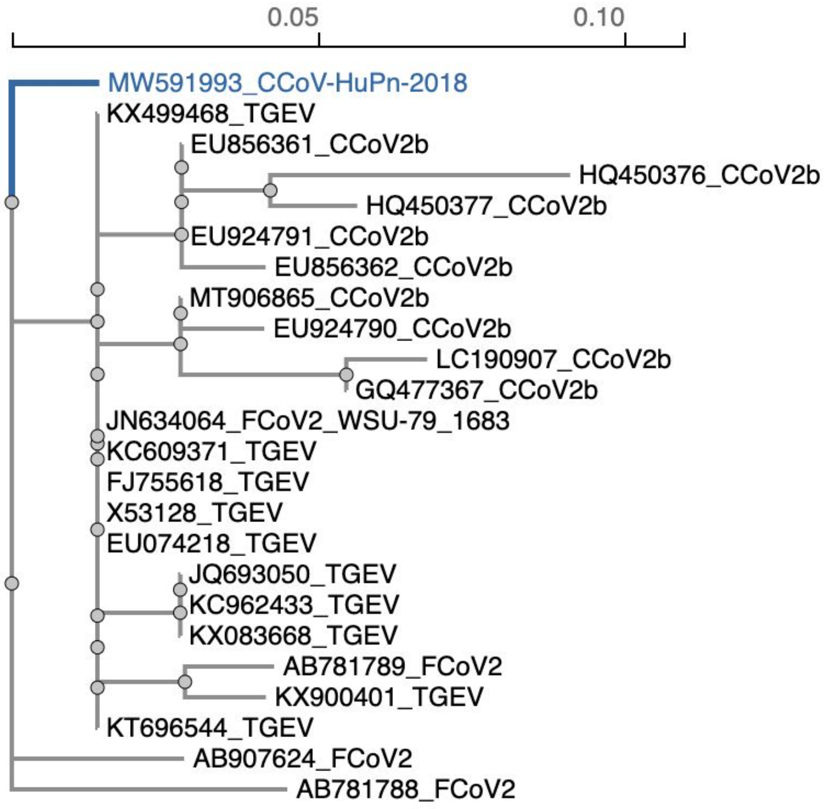
0.05
0.10
MW591993_CCoV-HuPn-2018
KX499468_TGEV
EU856361_CCoV2b
HQ450376_CCoV2b
HQ450377_CCoV2b
EU924791_CCoV2b
EU856362_CCoV2b
MT906865_CCoV2b
EU924790_CCoV2b
LC190907_CCoV2b
GQ477367_CCoV2b
JN634064_FCoV2_WSU-79_1683
KC609371_TGEV
FJ755618_TGEV
X53128_TGEV
EU074218_TGEV
JQ693050_TGEV
KC962433_TGEV
KX083668_TGEV
AB781789_FCoV2
KX900401_TGEV
KT696544_TGEV
AB907624_FCoV2
AB781788_FCoV2

6.

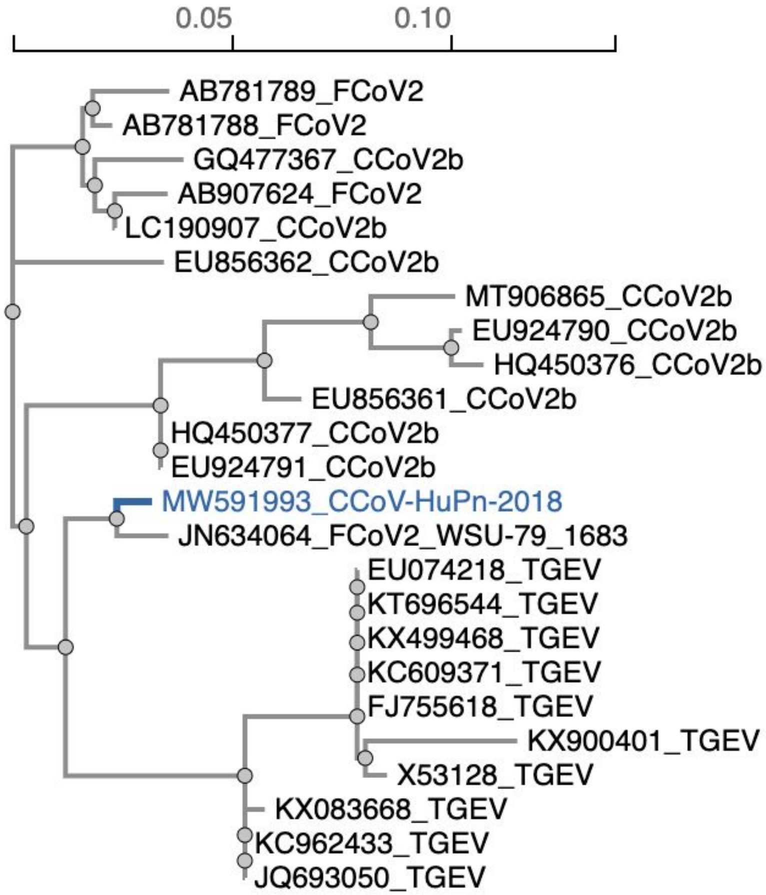
0.05
0.10
AB781789_FCoV2
AB781788_FCoV2
GQ477367_CCoV2b
AB907624_FCoV2
LC190907_CCoV2b
EU856362_CCoV2b
MT906865_CCoV2b
EU924790_CCoV2b
HQ450376_CCoV2b
EU856361_CCoV2b
HQ450377_CCoV2b
EU924791_CCoV2b
MW591993_CCoV-HuPn-2018
JN634064_FCoV2_WSU-79_1683
EU074218_TGEV
KT696544_TGEV
KX499468_TGEV
KC609371_TGEV
FJ755618_TGEV
KX900401_TGEV
X53128_TGEV
KX083668_TGEV
KC962433_TGEV
JQ693050_TGEV

7.

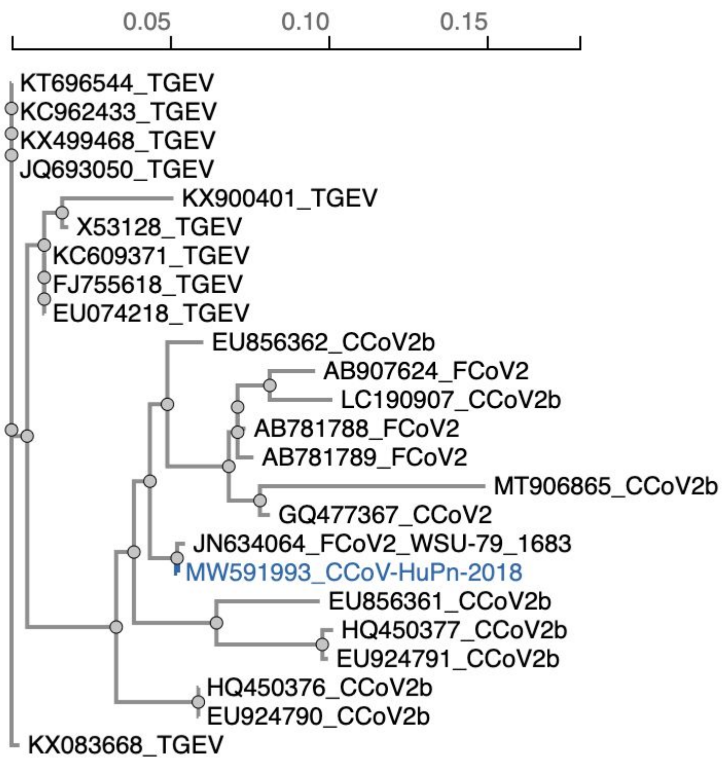
0.05
0.10
0.15
KT696544_TGEV
KC962433_TGEV
KX499468_TGEV
JQ693050_TGEV
KX900401_TGEV
X53128_TGEV
KC609371_TGEV
FJ755618_TGEV
EU074218_TGEV
EU856362_CCoV2b
AB907624_FCoV2
LC190907_CCoV2b
AB781788_FCoV2
AB781789_FCoV2
MT906865_CCoV2b
GQ477367_CCoV2
JN634064_FCoV2_WSU-79_1683
MW591993_CCoV-HuPn-2018
EU856361_CCoV2b
HQ450377_CCoV2b
EU924791_CCoV2b
HQ450376_CCoV2b
EU924790_CCoV2b
KX083668_TGEV

8.

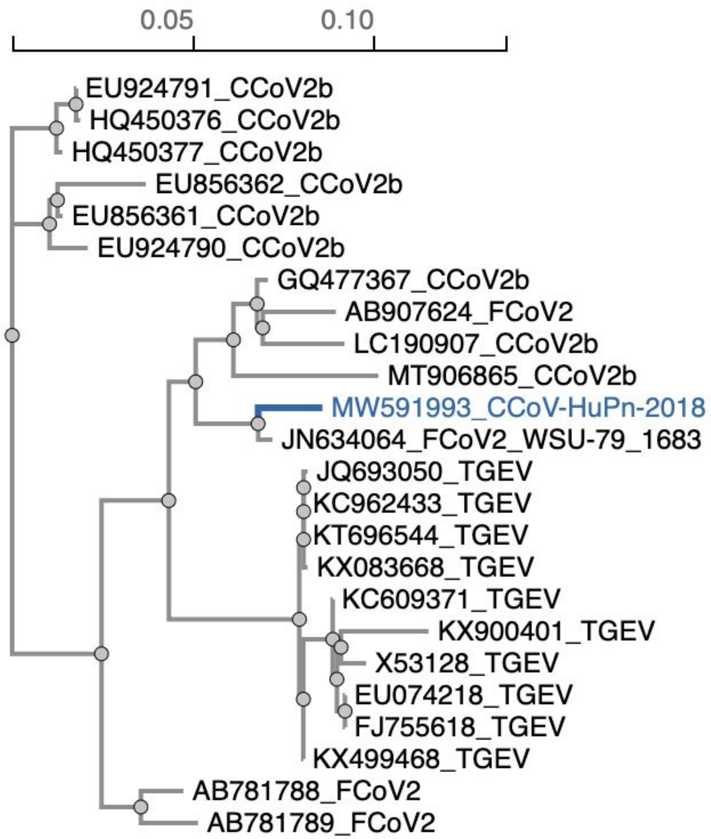
0.05
0.10
EU924791_CCoV2b
HQ450376_CCoV2b
HQ450377_CCoV2b
EU856362_CCoV2b
EU856361_CCoV2b
EU924790_CCoV2b
GQ477367_CCoV2b
AB907624_FCoV2
LC190907_CCoV2b
MT906865_CCoV2b
MW591993_CCoV-HuPn-2018
JN634064_FCoV2_WSU-79_1683
JQ693050_TGEV
KC962433_TGEV
KT696544_TGEV
KX083668_TGEV
KC609371_TGEV
KX900401_TGEV
X53128_TGEV
EU074218_TGEV
FJ755618_TGEV
KX499468_TGEV
AB781788_FCoV2
AB781789_FCoV2

9.

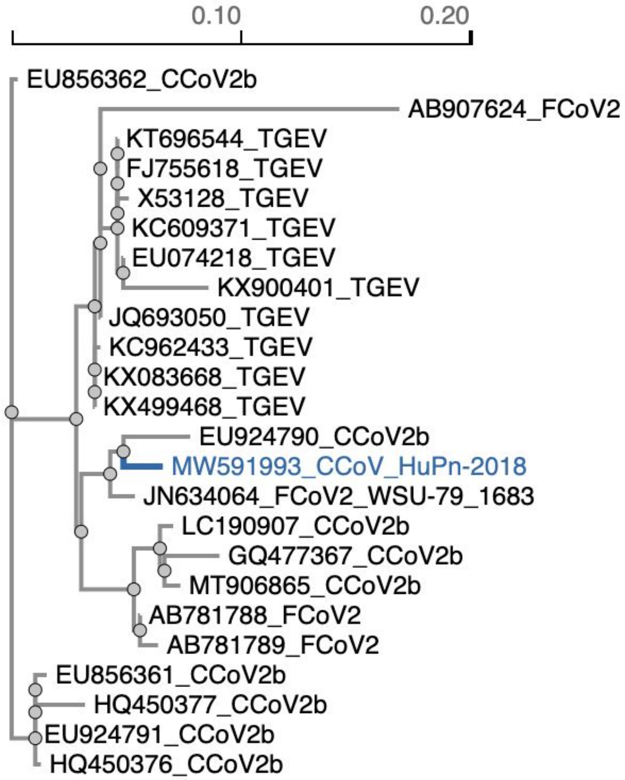

Figure S1. Phylogenetic trees for each GARD fragment inferred using RAxML. 1. The first non-recombinant fragment from alignment set I. The additional trees (2-9) correspond to non-recombinant fragments from alignment set II. Tree 2 corresponds to non-recombinant fragment 2 as depicted in the Fig. 1 gene map; tree 3 corresponds to non-recombinant fragment 3, and so on. These trees represent the phylogenetic incongruencies between the different non-recombinant fragments. The blue branch in each tree highlights CCoV-HuPn-2018.

Figure S2. TDR clock rate estimates

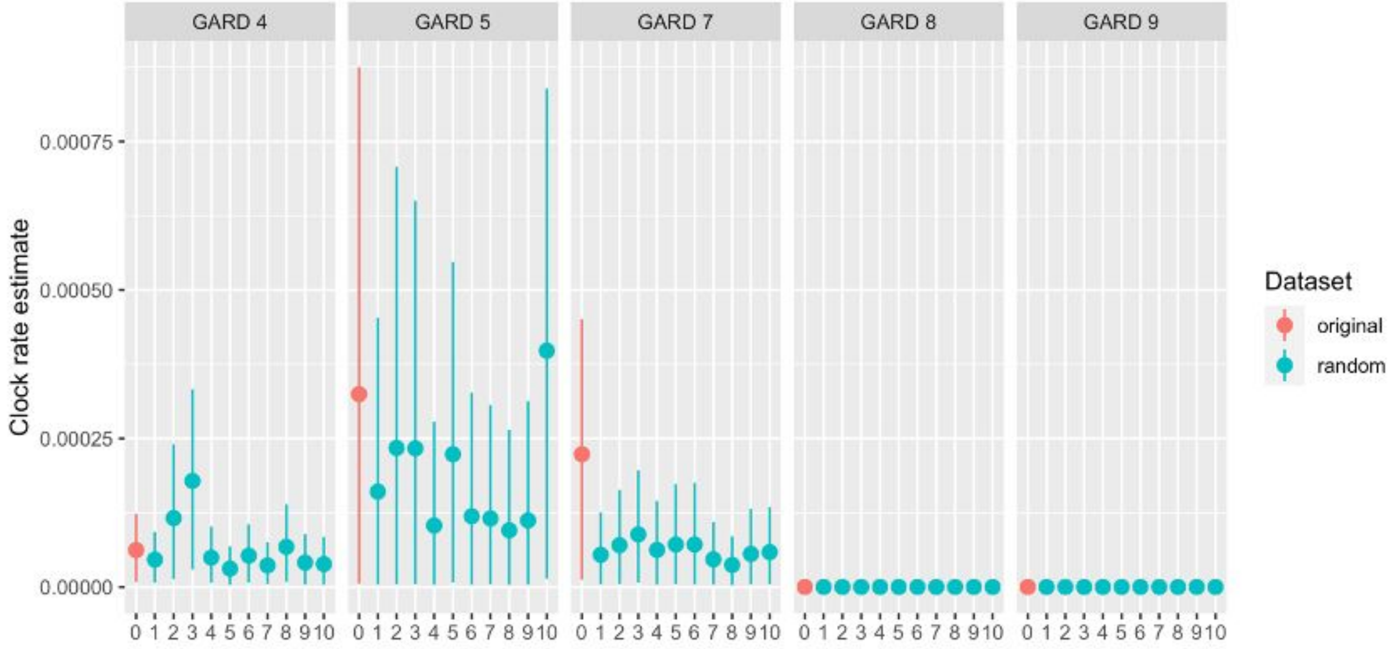

Figure S2. Mean and 95% HPD clock rate estimates for the original dataset and ten datasets with random dates are shown for the five GARD fragments with evidence of temporal signal on the root-tip regression analysis. Only GARD 7 had a mean clock rate estimate above the 95% HPD of the randomized datasets, indicating the presence of a temporal signal.

Table S3: Root-tip-regression results for each GARD partition

GARD partition	Correlation coefficient	R2
1	0.054	0.029
2	-0.046	0.0021
3	-0.11	0.013
4	0.2	0.04
5	0.2	0.039
6	0.088	0.0077
7	0.36	0.13
8	0.3	0.089
9	0.21	0.046

Figure S3. Ancestral host reconstruction and divergence time estimates

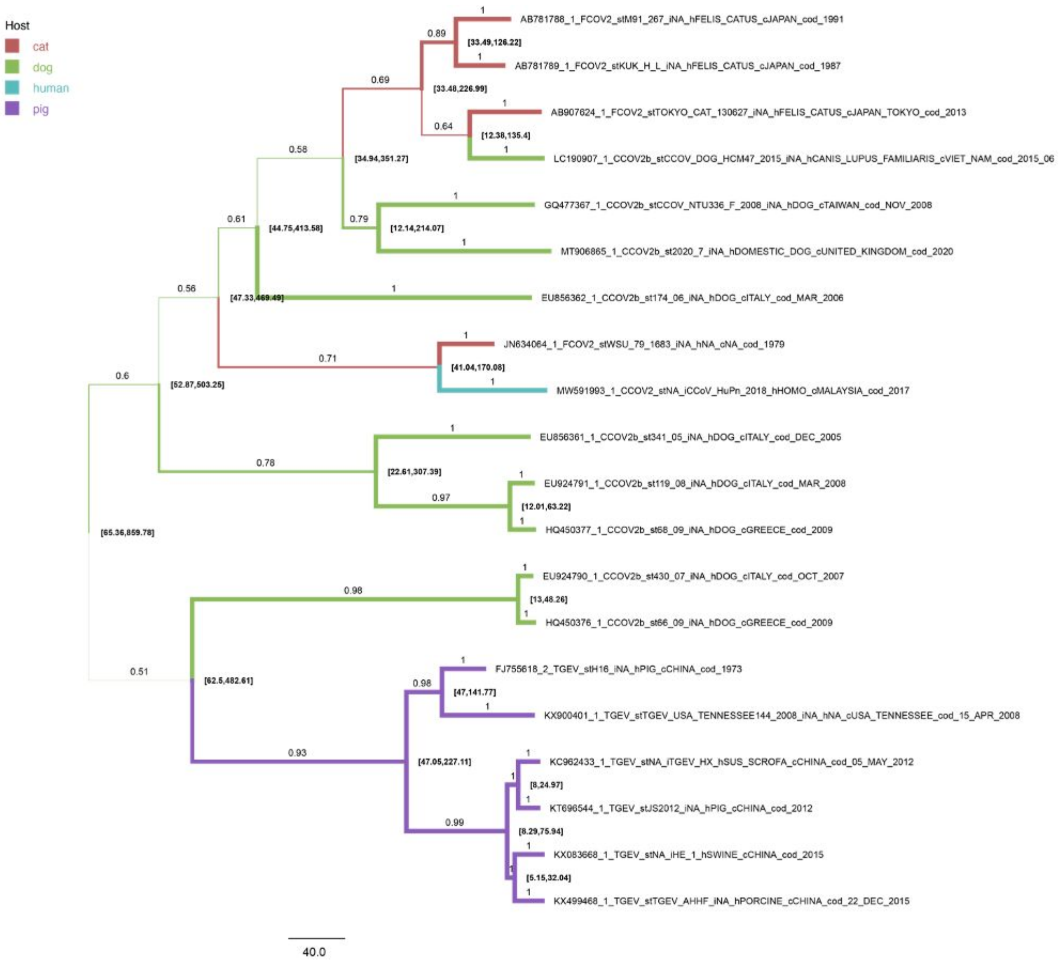

Figure S3. Ancestral host reconstruction and divergence time estimates**.** Branches are colored by inferred host species. The branch width is proportional to the posterior probability of host assignment (also labeled on branches). Internal nodes are labeled with the divergence time 95% HPD in years from the most recent sample date, 2017. CCoV-HuPn-2018 diverged from FCoV2 JN634064 between 40 and 170 years ago, with a median date estimate of 1957.

Figure S4. Positively selected and unique sites mapped to 0, A, and B domains using AlphaFold2 3D structural predictions

A

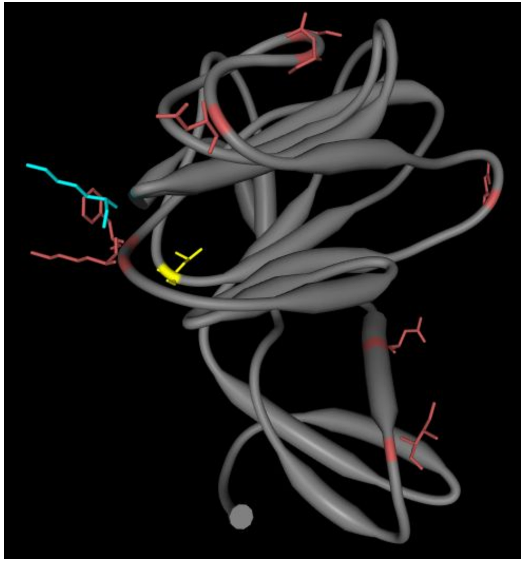

0-Domain

B

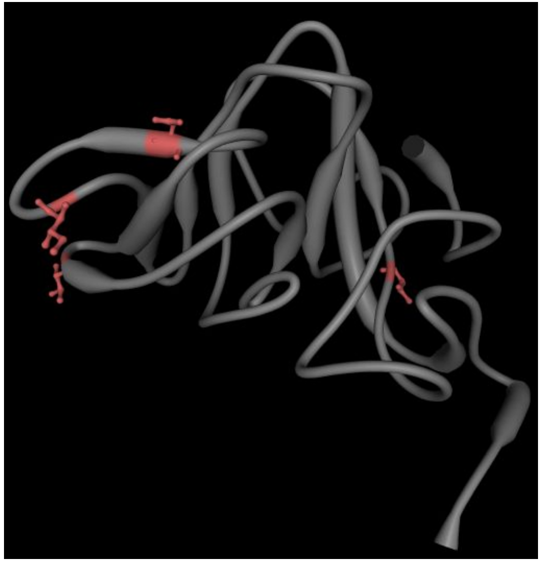

A-Domain

C

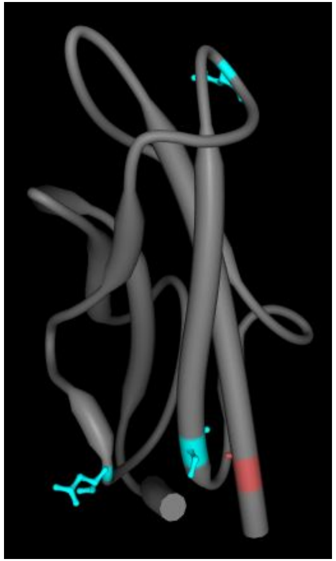

B-Domain

Figure S4: AlphaFold2 predicted 3D structures of 0-domain, A-domain, and B-domain (A), (B), and (C), respectively. When appropriate, positively selected sites, unique sites in CCoV-HuPn-2018 as compared to TGEV and CCoV2b sequences, and unique sites between CCoV-HuPn-2018 and HuCCoV_Z19Haiti are mapped to the structure in blue, red, and yellow, respectively. (A) There is one positively selected site in the 0-domain (blue), eight unique sites between CCoV-HuPn-2018 as compared to TGEV and CCoV2b sequences (red) and one site that is unique between CCoV-HuPn-2018 and HuCCoV_Z19Haiti (yellow). (B) The A domain has four unique sites in CCoV-HuPn-2018 as compared to TGEV and CCoV2b (red). There were neither positively selected sites, nor unique sites between CCoV-HuPn-2018 and HuCCoV_Z19Haiti in the A-domain. (C) There were three positively selected sites inferred within the B-domain (blue), where one site (I575) falls within the APN interacting residues (Y543/W586 [1]). There is one unique site in the B-domain of CCoV-HuPn-2018 as compared to TGEV and CCoV2b sequences. An interactive Observable Notebook for each domain can be found here: https://observablehq.com/d/80042b3f63cbb04e, https://observablehq.com/d/3e930a34f7b2c9c2, https://observablehq.com/d/1a683969c309cbe9 for 0-domain, A-domain, and B-domain, respectively.

Figure S5. Relaxed selection results for 0-domain

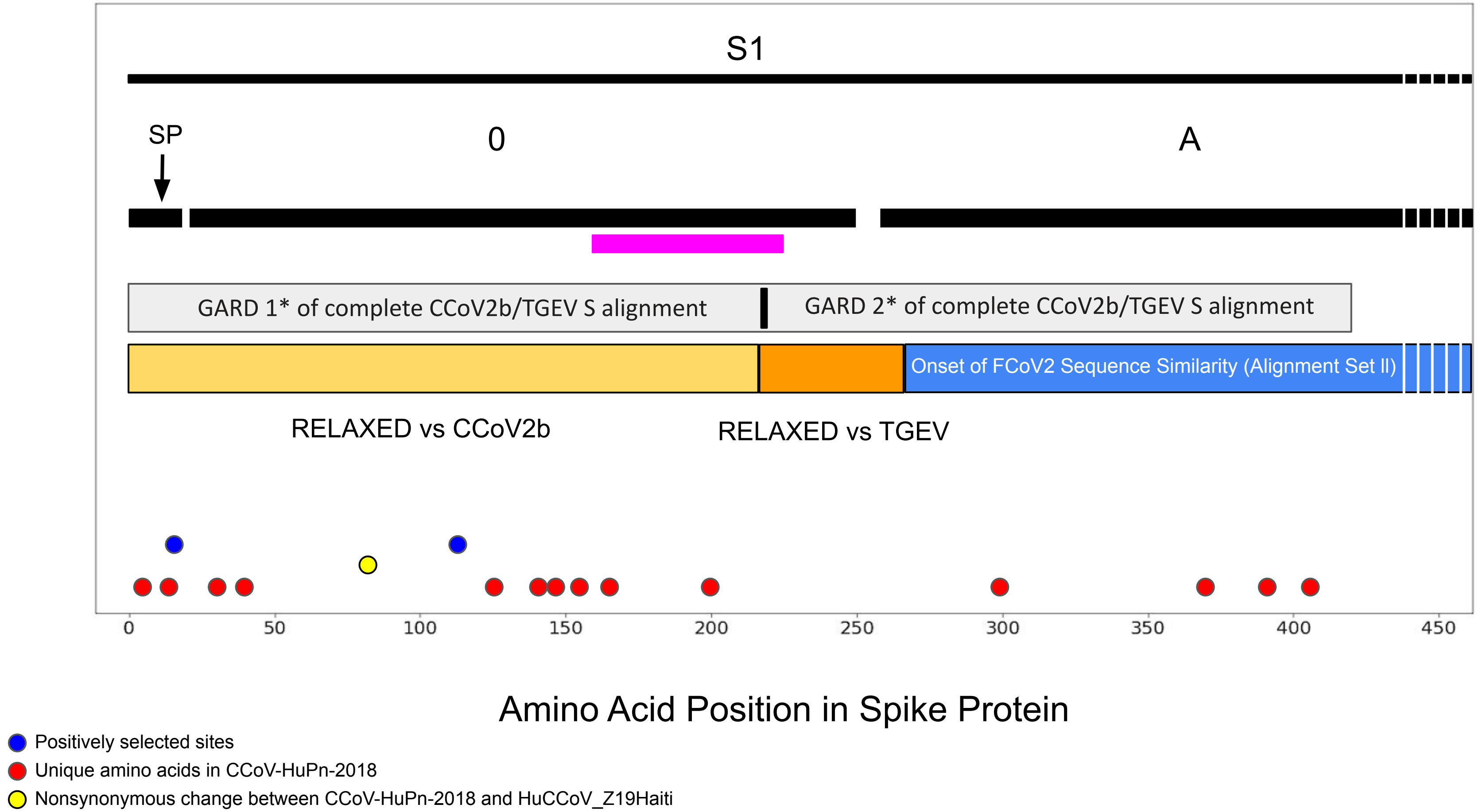

Figure S5. Relaxed selection results on exclusively CCoV2b/TGEV/CCoV-HuPn-2018 alignment for 0 and portion of A-domain. In analyses presented in the main text of the paper, alignment set I includes only the 0-domain, ending at onset of FCoV2 sequence similarity and beginning of alignment set II, and does not possess a significant GARD breakpoint. Alignment set I was significant for relaxed selection of CCoV-HuPn-2018 versus both CCoV2b and TGEV. In an alignment of exclusively CCoV2b/TGEV/CCoV-HuPn-2018 for the complete S gene there is a single GARD breakpoint - GARD 1* - and downstream of that is GARD 2* extending into the A-domain. Analysis of GARD 1* of this CCoV2b/TGEV/CCoV-HuPn-2018 S alignment is significant for relaxed selection of CCoV-HuPn-2018 against CCoV2b (yellow block; **K=0.09, p=0.005**). Analysis of GARD 2* of this CCoV2b/TGEV/CCoV-HuPn-2018 S alignment is not significant for relaxed selection; however, if one considers the block of sequence demarcating the beginning of GARD 2*, to the end of the 0-domain (orange block) and the beginning of alignment set II (blue block), and FCoV2 sequence similarity, this portion of the sequence is significant for relaxed selection of CCoV-HuPn-2018 against TGEV (**K=0.6, p=0.007**). The magenta box highlights the experimentally validated sialic acid binding region.
